# Supplementary material for: Crystal symmetry modification enables high-ranged in-plane thermoelectric performance in n-type SnSe crystals
Source: Nat Commun. 2025 Feb 20;16:1788. doi: 10.1038/s41467-025-57125-w (PMC11842792; doi:10.1038/s41467-025-57125-w)
Supplement: Supplementary file 1 — Supplementary Information [file 41467_2025_57125_MOESM1_ESM.pdf]

Supplementary Information

Crystal symmetry modification enables high-ranged in-plane thermoelectric performance in n-type SnSe crystals

Haonan Shi[1,2,3]†, Yi Wen[1]†, Shulin Bai[1], Cheng Chang[1], Lizhong Su[4], Tian Gao[1], Shibo Liu[1], Dongrui Liu[1], Bingchao Qin[1], Yongxin Qin[1], Huiqiang Liang[5], Xin Qian[5], Zhenghao Hou[6], Xiang Gao[7], Tianhang Zhou[8]*, Qing Tan[1]*, & Li-Dong Zhao[1,2,3]*

1 School of Materials Science and Engineering, Beihang University, Beijing 100191, China

2 Center for Bioinspired Science and Technology, Hangzhou International Innovation Institute, Beihang University, Hangzhou 311115, China

3 Tianmushan Laboratory, Beihang University, Hangzhou 311115, China

4 School of Materials Science and Engineering, Taiyuan University of Science and Technology, Taiyuan 030024, China

5 College of Physical Science and Technology, Hebei University, Baoding 071002, China

6 Shijiazhuang Key Laboratory of Low Carbon Energy Materials, College of Chemical Engineering, Shijiazhuang University, Shijiazhuang 050035, China

7 Center for High-Pressure Science and Technology Advanced Research (HPSTAR), Beijing 100094, China

8 College of Carbon Neutrality Future Technology, State Key Laboratory of Heavy Oil Processing, China University of Petroleum (Beijing), Beijing 102249, China

*Corresponding author Email: zhouth@cup.edu.cn; tanqing@ustb.edu.cn; zhaolidong@buaa.edu.cn

†: These authors contributed equally: Haonan Shi, Yi Wen

1. Calculations Details

1.1 Debye-Callaway model

The Callaway model is utilized to describe the effect of Te_{Se} and Mo_{Sn} point defects on the lattice thermal conductivity[1]. The ratio of the lattice thermal conductivities of the sample with point defects (κ_{lat}) and the parent material ($\kappa_{lat,p}$) can be expressed as[1,2]:

$$\frac{\kappa_{lat}}{\kappa_{lat,p}} = \frac{\arctan U}{U} \tag{S1}$$

$$U = \left(\frac{\pi^2 \theta_D V \kappa_{lat,p} \Gamma}{h v_a^2} \right)^{0.5} \tag{S2}$$

where θ_D is the Debye temperature, V is the average volume per atom, h is the Planck constant, and v_a is the sound velocity. Γ is the imperfection scaling parameter representing the scattering strength of the point defects, which is defined as:

$$\Gamma = \frac{1}{2} \cdot \left(\frac{M_{cation}}{M} \right)^2 \Gamma_{cation} + \frac{1}{2} \cdot \left(\frac{M_{anion}}{M} \right)^2 \Gamma_{anion} \tag{S3}$$

In this work, Te was first alloyed into SnSe and formed the Te_{Se} defects. Therefore, the imperfection scaling parameter on the cation sites (Γ_{cation}) can be regarded as zero, and the Equation (S3) can be written as:

$$\Gamma = \frac{1}{2} \cdot \left(\frac{M_{anion}}{M} \right)^2 \Gamma_{anion} \tag{S4}$$

where M_{anion}=(1-x)M_{Se}+xM_{Te}, representing the average mass of cation sites, M=(M_{Se}+M_{Te})/2, Γ_{anion} is the imperfection scaling parameter on the anion sites, which is defined as:

$$\Gamma_{anion} = \Gamma_{(Se,Te)} = \Gamma_S + \Gamma_M \tag{S5}$$

$$\Gamma_S = \varepsilon x(1-x) \left(\frac{\Delta r}{r_{(Se,Te)}} \right)^2 \tag{S6}$$

$$\Gamma_M = x(1-x) \left(\frac{\Delta M}{M_{(Se,Te)}} \right)^2 \tag{S7}$$

$$\varepsilon = \frac{2}{9}\left(\frac{6.4\gamma(1+\nu)}{1-\nu}\right)^2 \tag{S8}$$

$$\Delta r = r_{Te} - r_{Se} \tag{S9}$$

$$r_{(Se,Te)} = (1-x)r_{Se} + xr_{Te} \tag{S10}$$

$$\Delta M = M_{Te} - M_{Se} \tag{S11}$$

$$M_{(Se,Te)} = (1-x)M_{Se} + xM_{Te} \tag{S12}$$

where Γ_S and Γ_M are strain fluctuation and mass fluctuation, Δr and ΔM are the difference of radius and mass between impurity (Te) and host atoms(Se), r_i is the radius of the atom, M_i is the relative atomic mass of the atom, ε is a phenomenological adjustable parameter related to the Poisson ratio (ν) and Grüneisen parameter (γ), which are calculated by:

$$\nu = \frac{1-2\left(\frac{v_s}{v_l}\right)^2}{2-2\left(\frac{v_s}{v_l}\right)^2} \tag{S13}$$

$$\gamma = \frac{3}{2}\cdot\left(\frac{1+\nu}{2-3\nu}\right) \tag{S14}$$

where v_s and v_l are longitudinal and shear sound velocities, respectively.

Secondly, Mo was introduced to SnSe-Te. Here we regarded SnSe-Te as the matrix and mainly discussed the Mo_{Sn} defects. Thus, Γ_{anion} can be regarded as zero, and the Equation (S3) can be written as:

$$\Gamma = \frac{1}{2}\cdot\left(\frac{M_{cation}}{M}\right)^2 \Gamma_{cation} \tag{S15}$$

Then we replace Se and Te with Sn and Mo, and repeat the calculation from Equations S5 to S14.

1.2 Density functional theory (DFT) calculations

Density functional theory (DFT) calculations with projected augmented wave (PAW) pseudopotential formalism were performed within the Perdew-Burke-Ernzerhof

(PBE) exchange-correlation functional form of generalized gradient approximation (GGA) method as implemented in the Vienna Ab-initio Simulation Package (VASP) software[3-5]. The temperature-dependent electronic band structures were calculated by using the experimental structures at elevated temperatures (from 300 K to 773 K) deriving from the refined SR-XRD data. The plane-wave cutoff energy was set to 550 eV, and the 4*12*12 Monkhorst-Pack grids were used for the k-points sampling[6]. Based on SR-XRD data, the phonon band spectrum of SnSe and SnSe-Mo-Te at room temperature was calculated by density functional perturbation theory (DFPT) as implemented in PHONOPY code[7]. To ensure convergence, the supercell size is set to 2*4*4 (256 atoms), and the k-points are 1*2*2 in the Brillouin zone.

2. Supplementary Figures

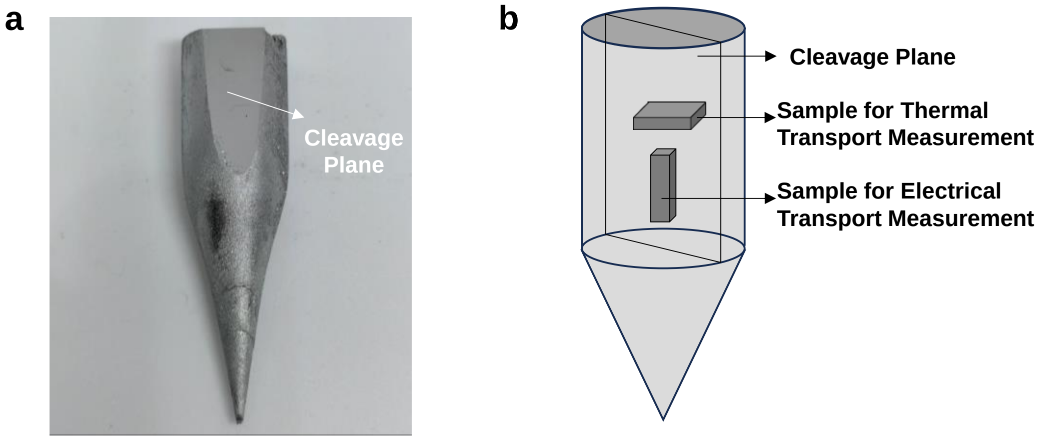

Fig. S1 The photo and schematic diagram of the SnSe crystal. a The photo of the SnSe crystal cleaved along (100) plane. **b** The schematic diagram of the SnSe crystal which shows how to cut out samples for thermoelectric transport measurement.

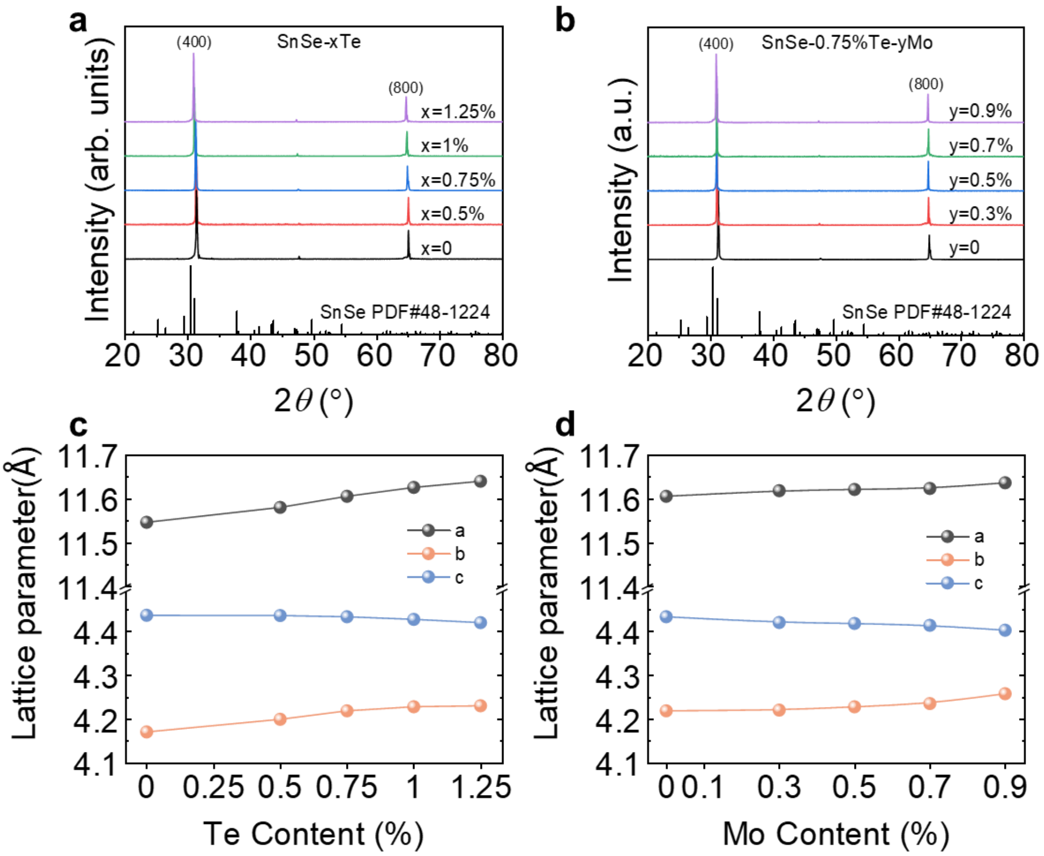

Fig. S2 X-ray diffraction patterns on the cleavage plane. a SnSe-*x*Te. **b** SnSe-0.75%Te-*y*Mo. **c** and **d** are the calculated lattice parameters of SnSe-*x*Te and SnSe-0.75%Te-*y*Mo, respectively.

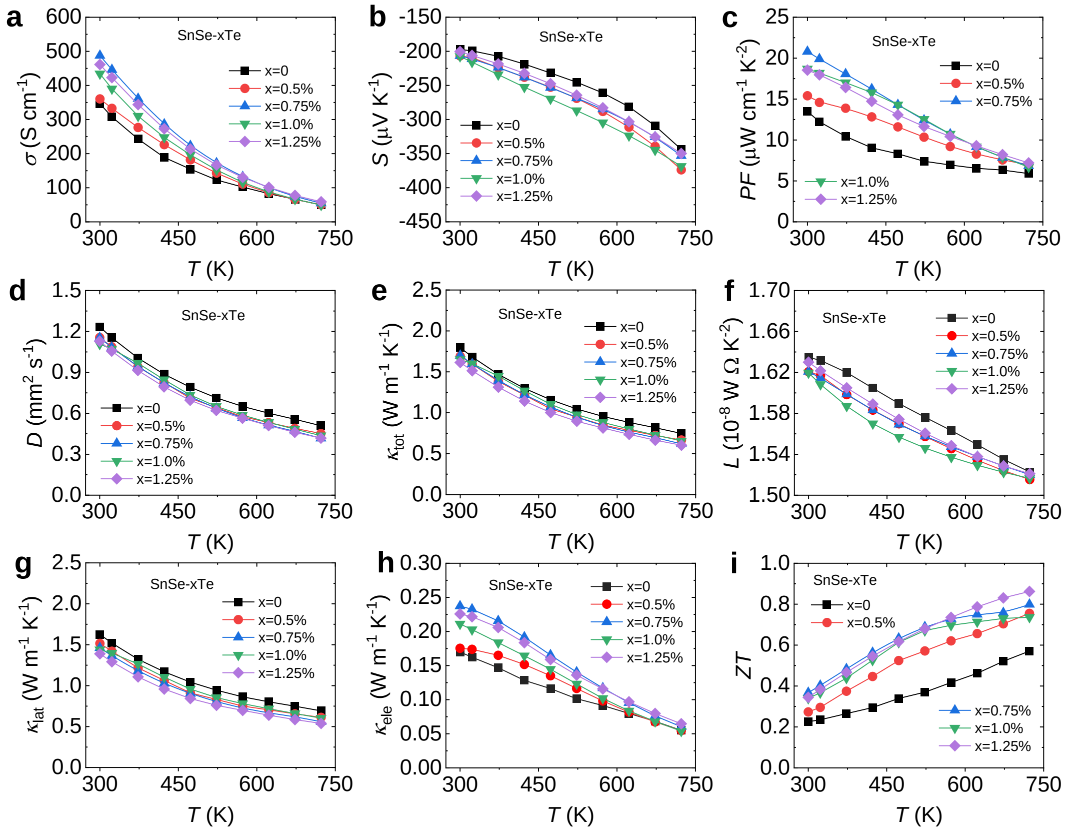

Fig. S3 The thermoelectric properties as functions of temperature in SnSe-*x*Te. a Electrical conductivity. **b** Seebeck coefficient. **c** Power factor. **d** Thermal diffusivity. **e** Total thermal conductivity. **f** Lorenz number. **g** Lattice thermal conductivity. **h** Electronic thermal conductivity. **i** *ZT* value.

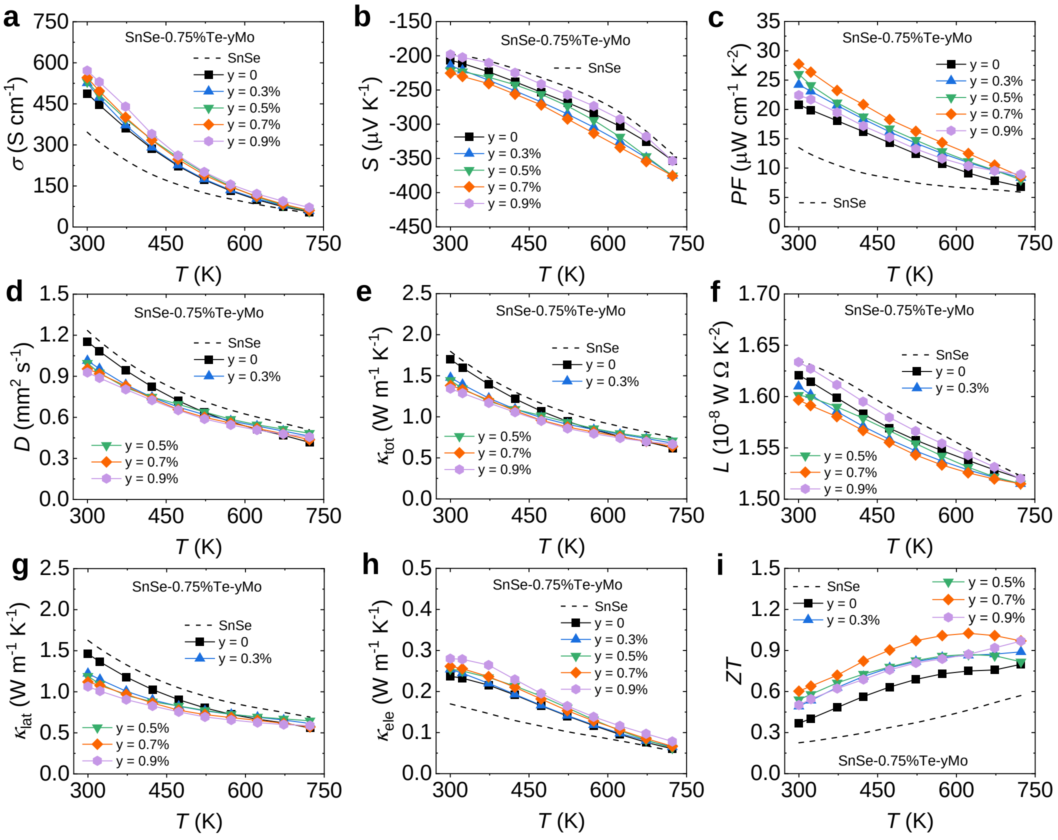

Fig. S4 The thermoelectric properties as functions of temperature in SnSe-0.75%Te-*y*Mo. **a** Electrical conductivity. **b** Seebeck coefficient. **c** Power factor. **d** Thermal diffusivity. **e** Total thermal conductivity. **f** Lorenz number. **g** Lattice thermal conductivity. **h** Electronic thermal conductivity. **i** *ZT* value.

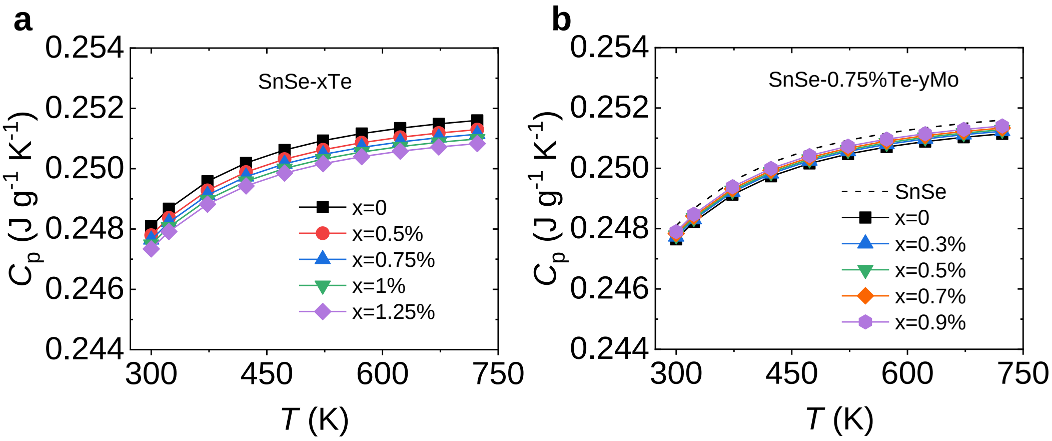

Fig. S5 The heat capacity (C_p) as the function of temperature. a SnSe-*x*Te and **b** SnSe-0.75%Te-*y*Mo.

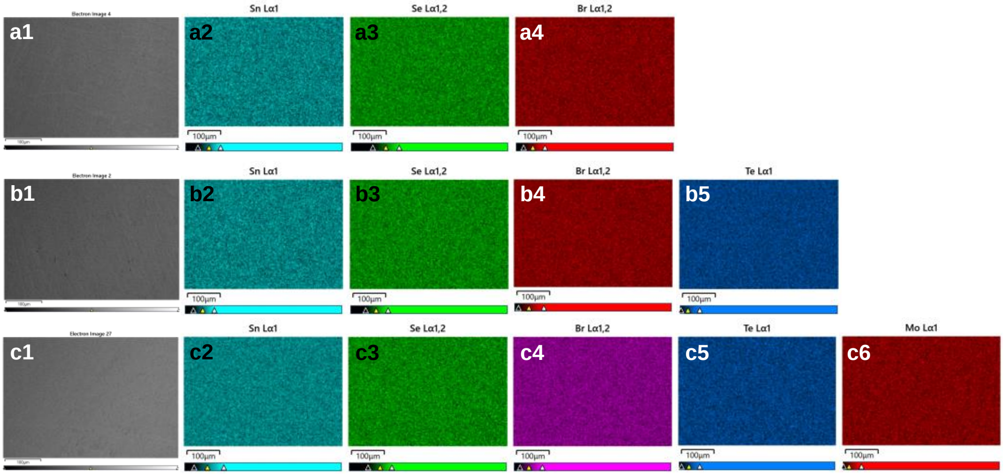

Fig. S6 Low-magnification scanning electron microscope (SEM) images and energy dispersive spectrometer (EDS) mapping images of different elements. a1 SEM image of SnSe and the corresponding elemental mappings for **a2** Sn, **a3** Se, and **a4** Br. **b1** SEM image of SnSe-0.75%Te and the corresponding elemental mappings for **b2** Sn, **b3** Se, **b4** Br, and **b5** Te. **c1** SEM image of SnSe-0.75%Te-0.7%Mo and the corresponding elemental mappings for **c2** Sn, **c3** Se, **c4** Br, **c5** Te, and **c6** Mo.

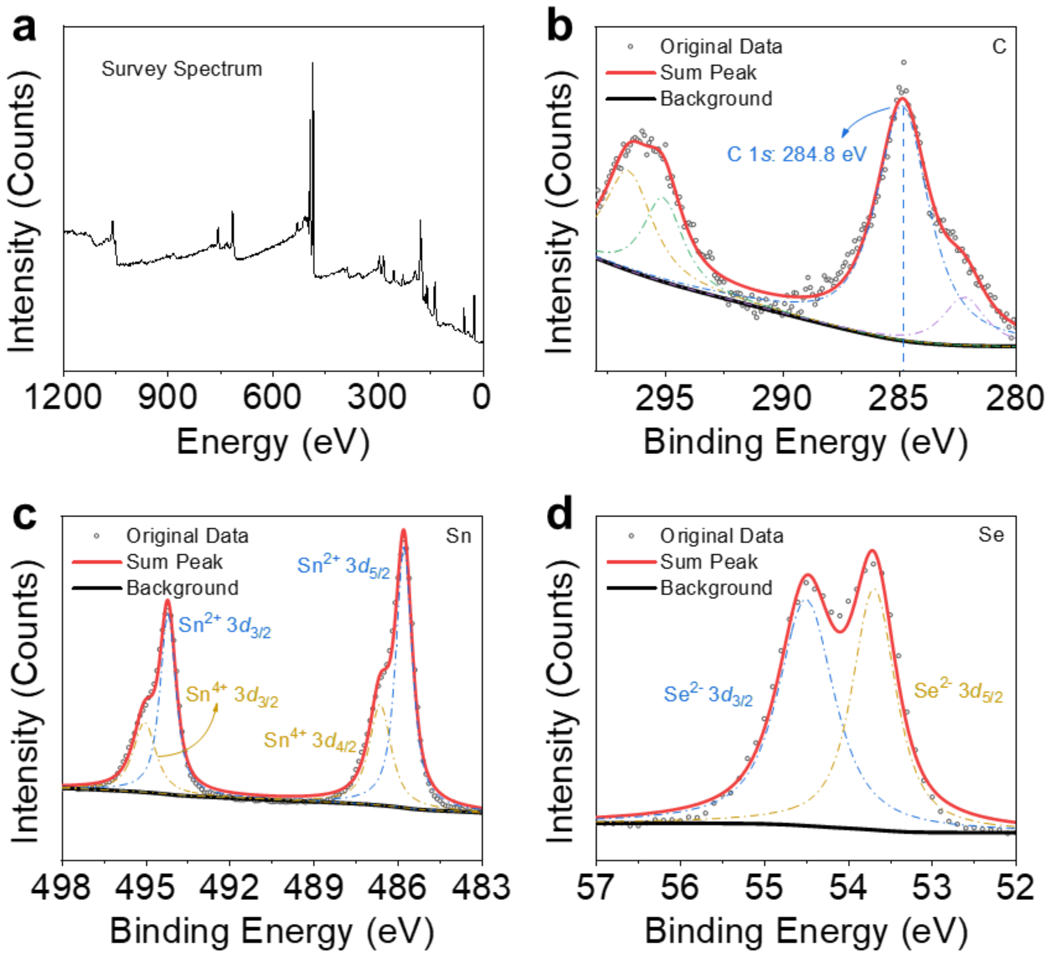

Fig. S7 X-ray photoelectron spectroscopy (XPS) spectra of SnSe-0.75%Te-0.7%Mo. a Survey spectrum. **b** C 1*s*. **c** Sn 3*d*. **d** Se 3*d*.

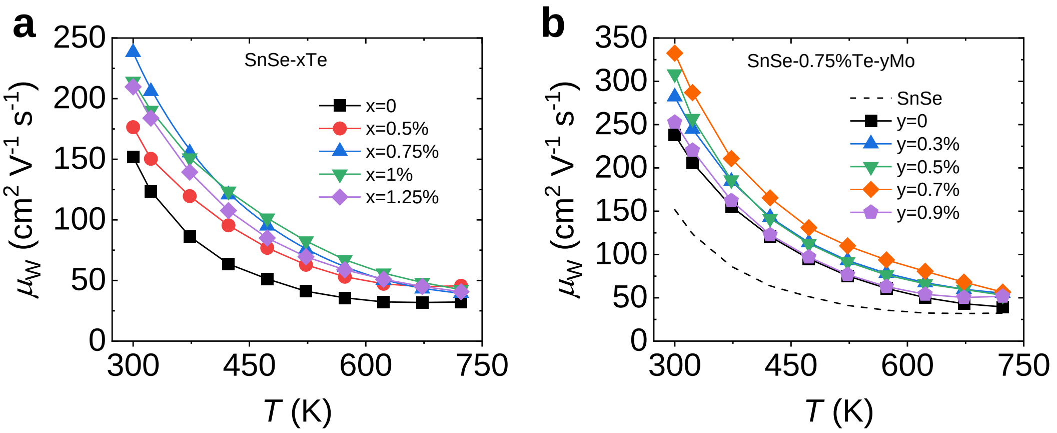

Fig. S8 The weighted mobility (*μ*W) as a function of temperatures. a SnSe-*x*Te. b SnSe-0.75%Te-*y*Mo.

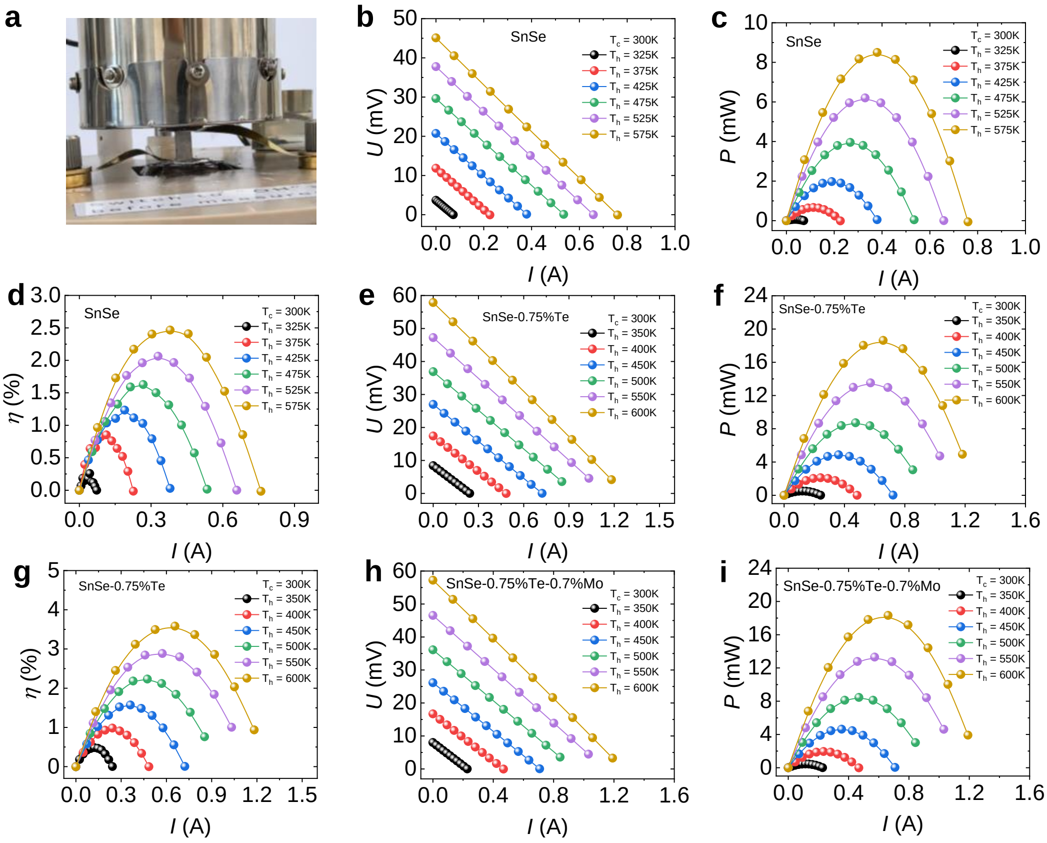

Fig. S9 The conversion efficiency measurements of the single-leg thermoelectric devices. a Photo from the experimental setup for the single-leg device by Mini-PEM. **b** Output Voltage, **c** Output power, and **d** Conversion efficiency as functions of current in SnSe. **e** Output Voltage, **f** Output power, and **g** Conversion efficiency as functions of current in SnSe-0.75%Te. **h** Output Voltage and **i** Output power as functions of current in SnSe-0.75%Te-0.7%Mo.

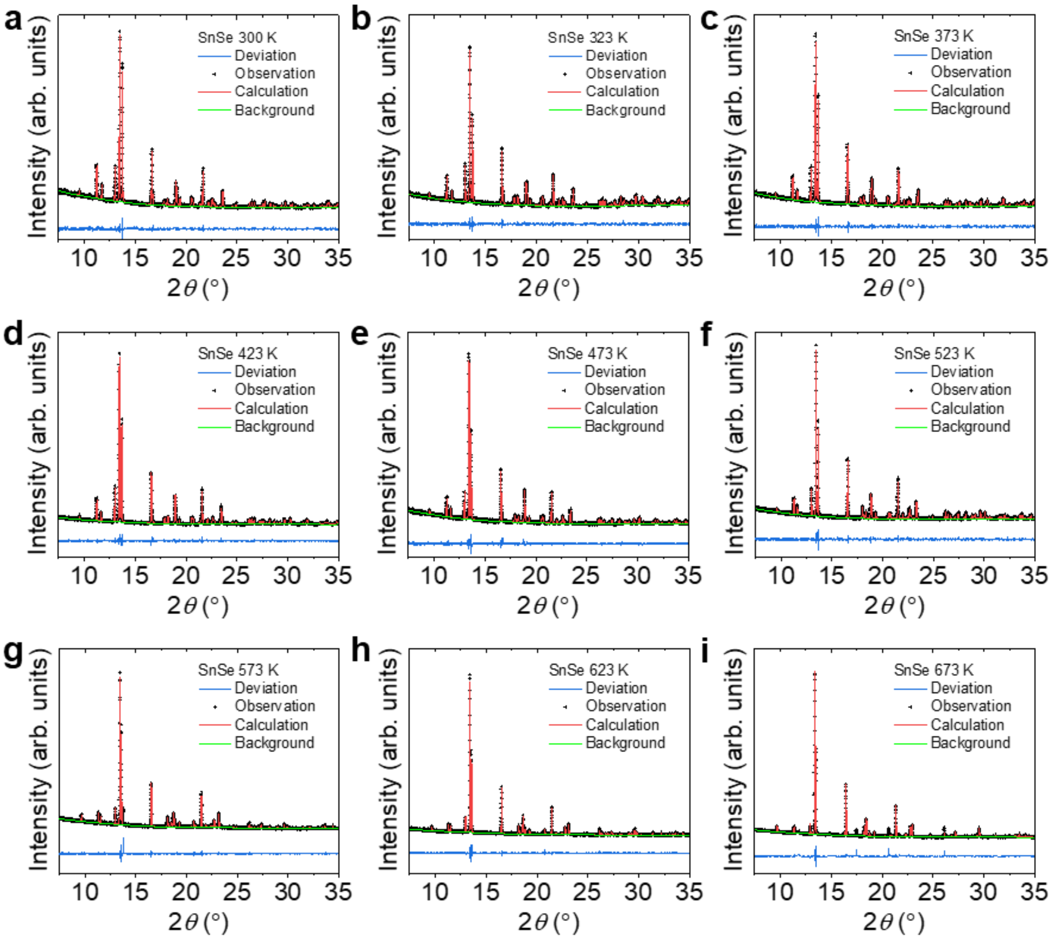

Fig. S10 SR-XRD patterns and Rietveld refinements of SnSe at different temperatures. a 300 K. **b** 323 K. **c** 373 K. **d** 423 K. **e** 473 K. **f** 523 K. **g** 573 K. **h** 623 K. **i** 673 K.

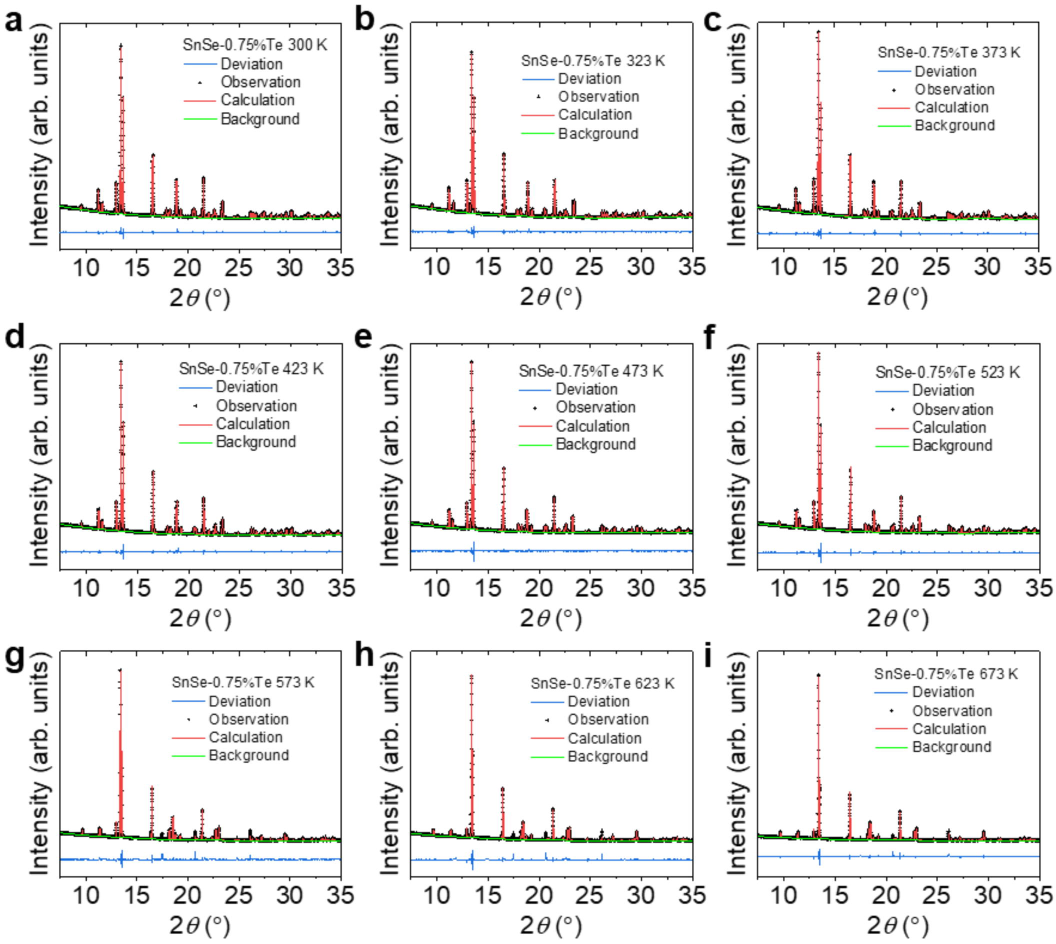

Fig. S11 SR-XRD patterns and Rietveld refinements of SnSe-0.75%Te at different temperatures. a 300 K. **b** 323 K. **c** 373 K. **d** 423 K. **e** 473 K. **f** 523 K. **g** 573 K. **h** 623 K. **i** 673 K.

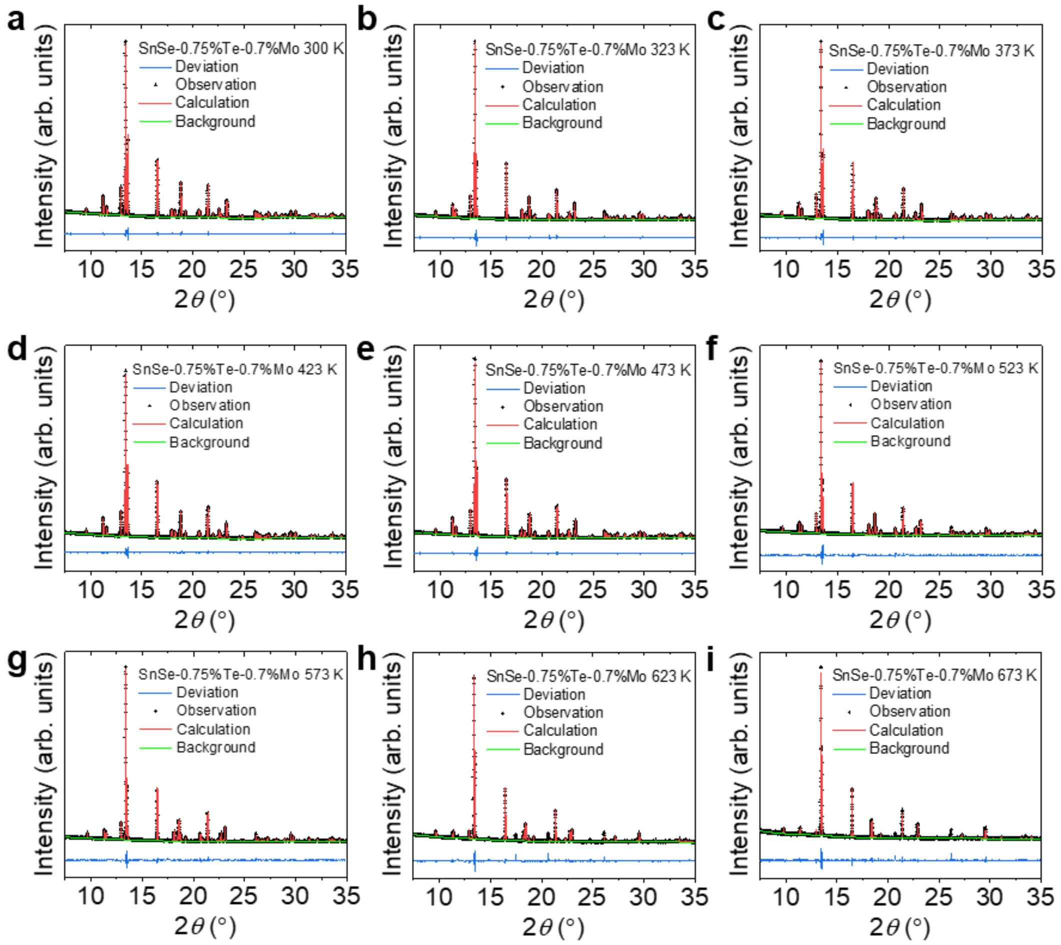

Fig. S12 SR-XRD patterns and Rietveld refinements of SnSe-0.75%Te-0.7%Mo at different temperatures. a 300 K. **b** 323 K. **c** 373 K. **d** 423 K. **e** 473 K. **f** 523 K. **g** 573 K. **h** 623 K. **i** 673 K.

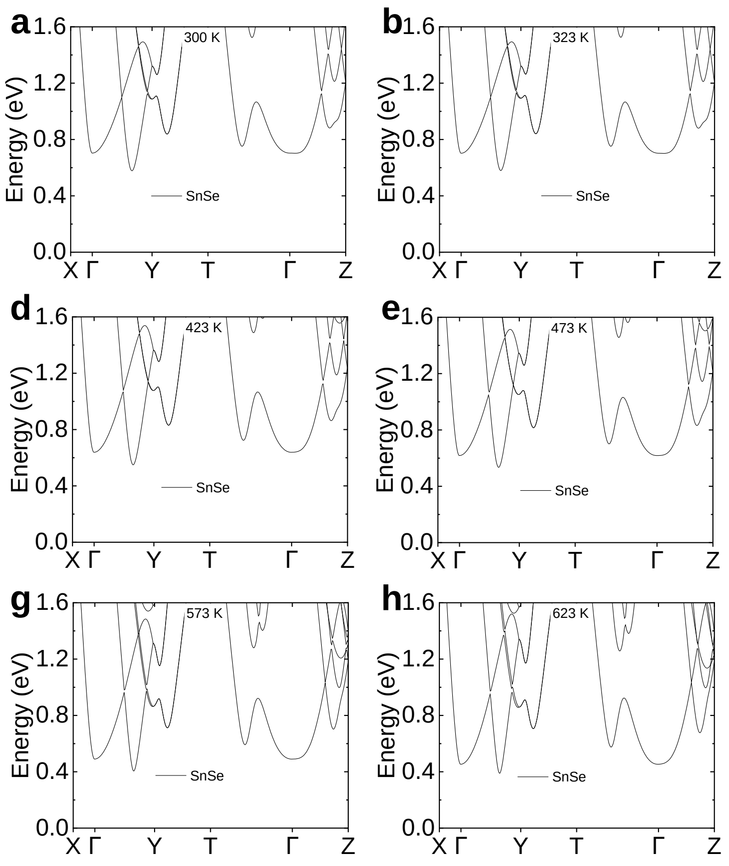

Fig. S13 Calculated electronic band structure of SnSe based on the refined structure from SR-XRD at different temperatures. a 300 K. **b** 323 K. **c** 373 K. **d** 423 K. **e** 473 K. **f** 523 K. **g** 573 K. **h** 623 K. **i** 673 K.

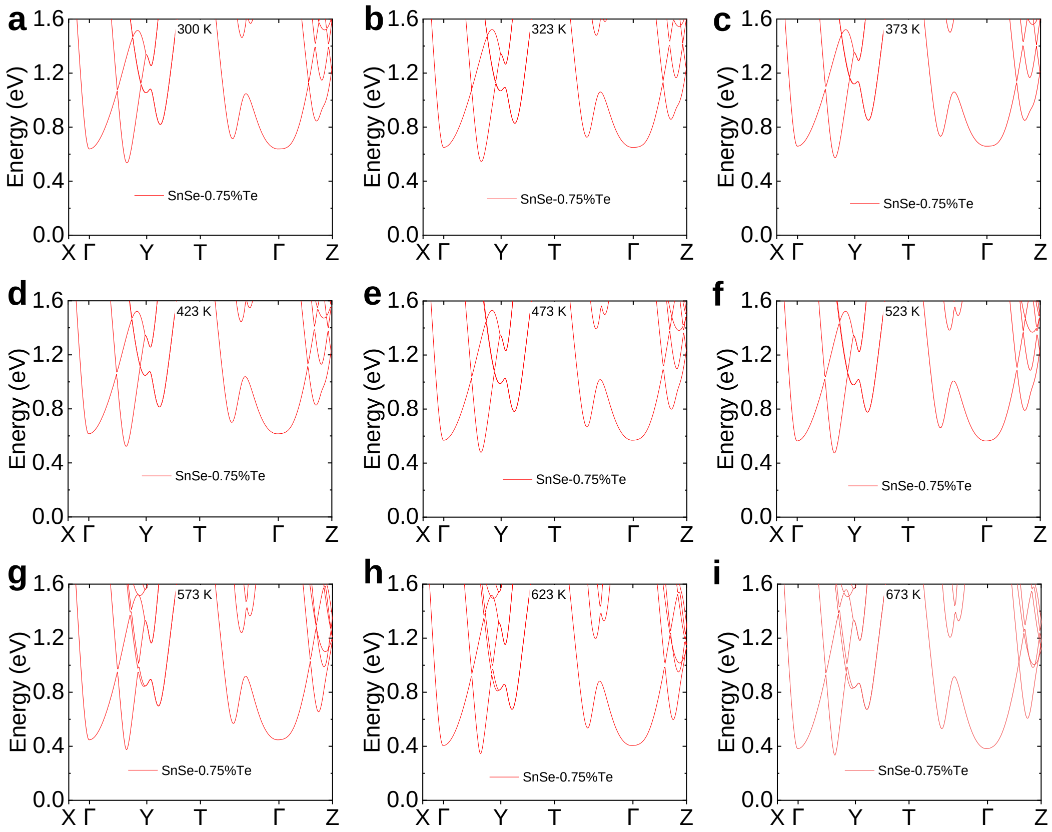

Fig. S14 Calculated electronic band structure of SnSe-0.75%Te based on the refined structure from SR-XRD at different temperatures. **a** 300 K. **b** 323 K. **c** 373 K. **d** 423 K. **e** 473 K. **f** 523 K. **g** 573 K. **h** 623 K. **i** 673 K.

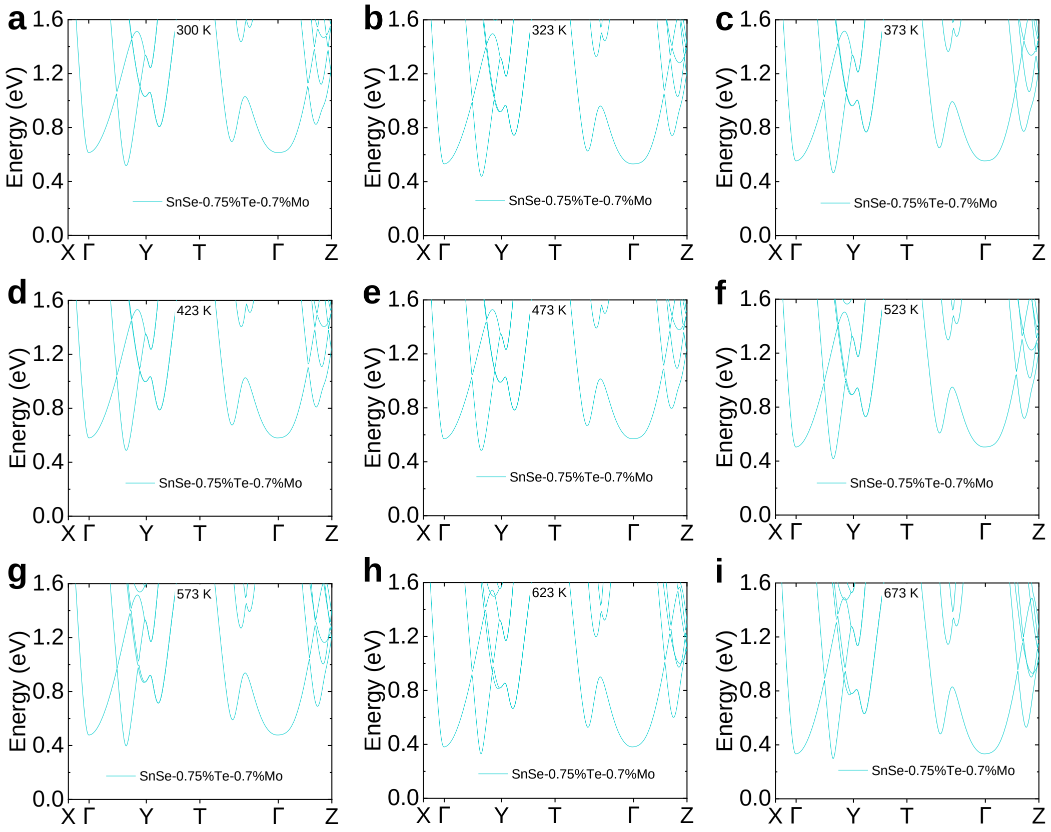

Fig. S15 Calculated electronic band structure of SnSe-0.75%Te-0.7%Mo based on the refined structure from SR-XRD at different temperatures. a 300 K. **b** 323 K. **c** 373 K. **d** 423 K. **e** 473 K. **f** 523 K. **g** 573 K. **h** 623 K. **i** 673 K.

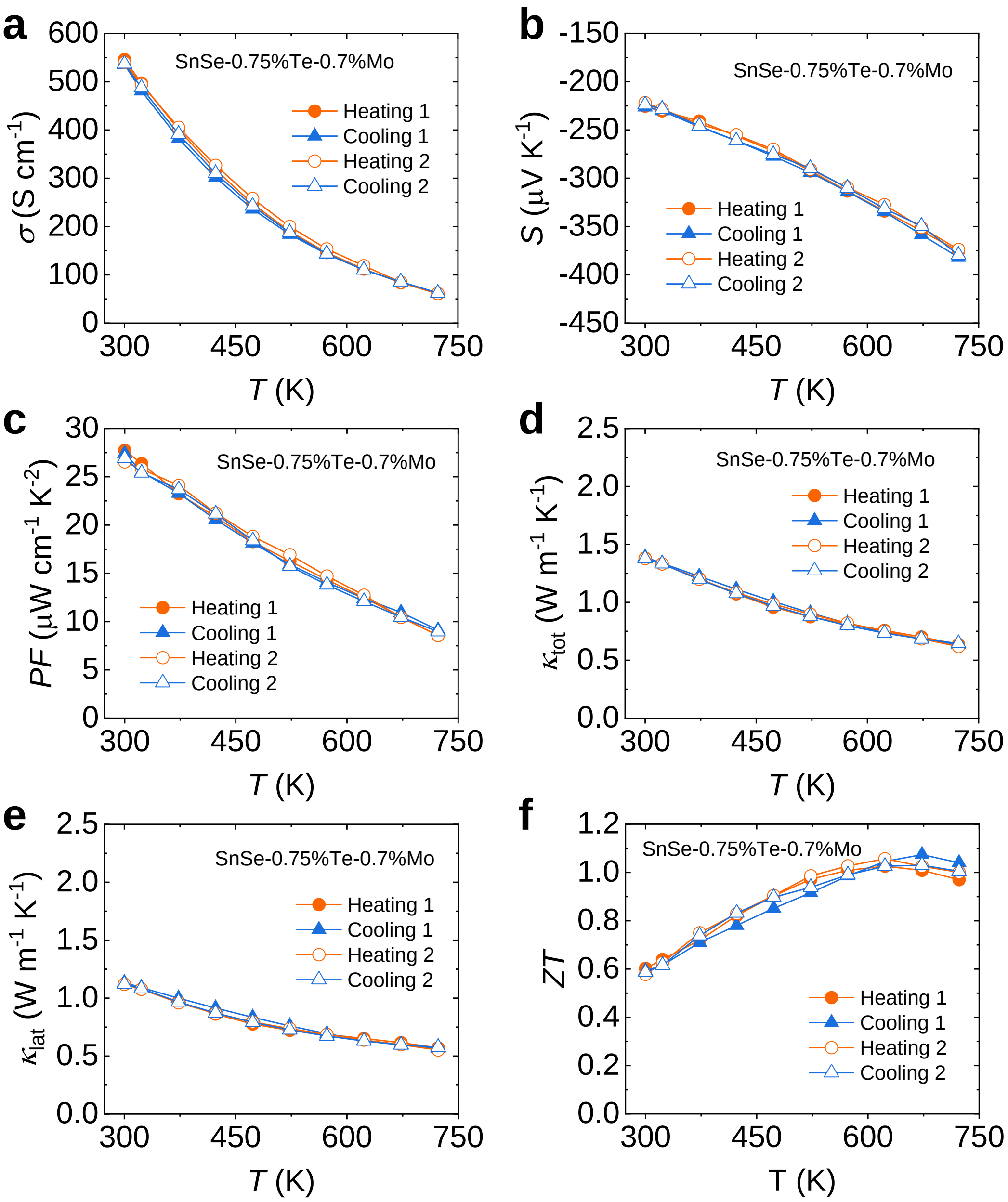

Fig. S16 The thermal stability of thermoelectric properties in SnSe-0.75%Te-*y*Mo. **a** Electrical conductivity. **b** Seebeck coefficient. **c** Power factor. **d** Total thermal conductivity. **e** Lattice thermal conductivity **f** *ZT* value.

3. Supplementary Tables

Table S1 The sound velocity of $Sn_{1-y}Mo_ySe_{0.97-x}Te_xBr_{0.03}$ (x = 0, 0.5%, 0.75%, 1%, 1.25%, and y = 0, 0.3%, 0.5%, 0.7% 0.9%).

Sample	Longitudinal sound velocity ($m\ s^{-1}$)	Shear sound velocity ($m\ s^{-1}$)
x = 0, y = 0	4487.18	2184.12
x = 0.5%, y = 0	3717.39	2220.78
x = 0.75%, y = 0	3978.72	2200.00
x = 1%, y = 0	3806.45	2125.19
x = 1.25%, y = 0	3756.10	2087.09
x = 0.75%, y = 0.3%	3634.15	1935.06
x = 0.75%, y = 0.5%	3829.41	1881.35
x = 0.75%, y = 0.7%	3917.74	1921.82
x = 0.75%, y = 0.9%	3564.15	1911.06

Table S2 The detailed refinement results of SnSe crystal with the temperature from 300 K to 723 K.

T (K)	Sn					Se				
	x	*y*	*z*	Occ	Uiso	*x*	*y*	*z*	Occ	Uiso
300	0.12059	0.25	0.10008	1.015	0.03	0.85606	0.25	0.49092	0.953	0.023
323	0.12054	0.25	0.09999	0.997	0.028	0.85601	0.25	0.49083	1.008	0.022
373	0.11989	0.25	0.09714	1.000	0.029	0.85536	0.25	0.48799	1.004	0.022
423	0.11922	0.25	0.09575	1.004	0.025	0.85469	0.25	0.48660	1.007	0.018
473	0.11982	0.25	0.09403	1.003	0.026	0.85529	0.25	0.48488	0.996	0.020
523	0.11879	0.25	0.08438	0.990	0.023	0.85426	0.25	0.47523	1.019	0.016
573	0.12082	0.25	0.08031	0.996	0.027	0.85629	0.25	0.47116	1.025	0.020
623	0.12026	0.25	0.07596	0.997	0.025	0.85573	0.25	0.46681	0.999	0.019
673	0.11963	0.25	0.06570	1.006	0.020	0.85509	0.25	0.45650	0.974	0.013
723	0.11968	0.25	0.06456	1.007	0.018	0.85514	0.25	0.45660	0.972	0.012

Table S3 The detailed refinement results of SnSe-0.75%Te crystal with the temperature from 300 K to 723 K.

T **(K)**	**Sn**					**Se**				
	x	***y***	***z***	**Occ**	**Uiso**	***x***	***y***	***z***	**Occ**	**Uiso**
300	0.11985	0.25	0.09425	0.994	0.026	0.85531	0.25	0.48509	1.011	0.020
323	0.11982	0.25	0.09523	0.995	0.028	0.85524	0.25	0.48607	1.010	0.021
373	0.11970	0.25	0.09642	1.009	0.030	0.85516	0.25	0.48726	1.007	0.023
423	0.11957	0.25	0.09230	0.999	0.027	0.85504	0.25	0.48314	1.016	0.020
473	0.11944	0.25	0.08827	0.989	0.028	0.85490	0.25	0.47911	1.020	0.022
523	0.11964	0.25	0.08777	0.997	0.030	0.85510	0.25	0.47861	1.006	0.023
573	0.12044	0.25	0.07309	0.999	0.023	0.85590	0.25	0.46393	0.982	0.016
623	0.12075	0.25	0.06778	0.994	0.022	0.85621	0.25	0.45862	0.991	0.015
673	0.11951	0.25	0.06604	1.026	0.018	0.85498	0.25	0.45688	1.013	0.011
723	0.12079	0.25	0.06120	0.980	0.018	0.85625	0.25	0.45200	1.031	0.011

Table S4 The detailed refinement results of SnSe-0.75%Te-0.7%Mo crystal with the temperature from 300 K to 723 K.

T **(K)**	**Sn**					**Se**				
	x	***y***	***z***	**Occ**	**Uiso**	***x***	***y***	***z***	**Occ**	**Uiso**
300	0.11990	0.25	0.09221	1.024	0.030	0.85537	0.25	0.48305	1.058	0.024
323	0.12046	0.25	0.08389	1.022	0.025	0.85593	0.25	0.47473	1.046	0.018
373	0.11990	0.25	0.08649	1.025	0.027	0.85537	0.25	0.47733	1.046	0.027
423	0.11941	0.25	0.08932	1.024	0.029	0.85488	0.25	0.48016	1.058	0.022
473	0.11957	0.25	0.08833	1.020	0.027	0.85504	0.25	0.47917	1.060	0.020
523	0.12039	0.25	0.08087	1.026	0.023	0.85586	0.25	0.47171	1.032	0.017
573	0.12032	0.25	0.07730	1.028	0.023	0.85579	0.25	0.46814	1.022	0.016
623	0.11951	0.25	0.06604	1.026	0.018	0.85498	0.25	0.45688	1.022	0.011
673	0.12072	0.25	0.06230	1.003	0.019	0.85618	0.25	0.45310	0.987	0.012
723	0.12009	0.25	0.06250	0.973	0.021	0.8555	0.25	0.45330	1.062	0.014

Table S5 The comparison of the electrical conductivity and carrier mobility along the two directions at room temperature. The σ_{in}, σ_{out}, μ_{in}, and μ_{out} are the electrical conductivity and carrier mobility along the in-plane and out-of-plane, respectively.

Sample	σ_{in} (S cm^{-1})	μ_{in} (cm^2 V^{-1} s^{-1})	σ_{out} (S cm^{-1})	μ_{out} (cm^2 V^{-1} s^{-1})
SnSe	346.39	247.42	139.55	99.68
SnSe-0.75%Te	487.60	277.05	171.79	97.61
SnSe-0.75%Te-0.7%Mo	545.81	422.20	239.91	185

Supplementary Reference

1 Callaway, J. & Baeyer, H. C. V. Effect of point imperfections on lattice thermal conductivity. *Phys. Rev.* **120**, 1149-1154 (1960).

2 Wan, C. L. *et al.* Effect of point defects on the thermal transport properties of $(La_xGd_{1-x})_2Zr_2O_7$: experiment and theoretical model. *Phys. Rev. B* **74**, 144109 (2006).

3 Hafner, J. Ab-initio simulations of materials using VASP: Density-functional theory and beyond. *J. Comput. Chem.* **29**, 2044-2078 (2008).

4 Perdew, J. P., Burke, K. & Ernzerhof, M. Generalized gradient approximation made simple. *Phys. Rev. Lett.* **77**, 3865-3868 (1996).

5 Blochl, P. E. Projector augmented-wave method. *Phys. Rev. B* **50**, 17953-17979 (1994).

6 Monkhorst, H. J. & Pack, J. D. Special points for Brillouin-zone integrations. *Phys. Rev. B* **13**, 5188-5192 (1976).

7 Togo, A. & Tanaka, I. First principles phonon calculations in materials science. *Scr. Mater.* **108**, 1-5 (2015).
